# Chlorogenic Acid Attenuates Oxidative Stress-Induced Intestinal Epithelium Injury by Co-Regulating the PI3K/Akt and IκBα/NF-κB Signaling

**DOI:** 10.3390/antiox10121915

**Published:** 2021-11-29

**Authors:** Jiali Chen, Yuheng Luo, Yan Li, Daiwen Chen, Bing Yu, Jun He

**Affiliations:** 1 Institute of Animal Nutrition, Sichuan Agricultural University, Chengdu 611130, China; li_yang@sdau.edu.cn (J.C.); yhluo@sicau.edu.cn (Y.L.); 13987@sicau.edu.cn (Y.L.); dwchen@sicau.edu.cn (D.C.); 12824@sicau.edu.cn (B.Y.); 2 Key Laboratory of Animal Disease-Resistance Nutrition of the Ministry of Agriculture, Chengdu 611130, China; 3 Guilin Fengpeng Bio-Tech Co., Ltd., Guilin 541199, China

**Keywords:** chlorogenic acid, weaned pigs, intestinal epithelial cells, oxidative stress, inflammation, signaling pathway

## Abstract

Chlorogenic acid (CGA) is a natural polyphenol compound abundant in green plants with antioxidant and anti-inflammatory activities. Here, we explore its protective effects and potential mechanisms of action on intestinal epithelium exposure to oxidative stress (OS). We show that CGA attenuated OS-induced intestinal inflammation and injury in weaned pigs, which is associated with elevated antioxidant capacity and decreases in inflammatory cytokine secretion and cell apoptosis. In vitro study showed that CGA elevated phosphorylation of two critical signaling proteins of the phosphatidylinositol-3-kinase (PI3K)/protein kinase B (Akt) pathway, Akt and nuclear factor erythroid-derived-related factor 2, leading to the elevated expression of intracellular antioxidant enzymes and heme oxygenase-1 (HO-1). Specific inhibition of HO-1 partially abolished its anti-inflammatory effect in IPEC-J2 cells exposure to OS. Interestingly, CGA suppressed the tumor necrosis factor-α (TNF-α) induced inflammatory responses in IPEC-J2 cells by decreasing phosphorylation of two critical inflammatory signaling proteins, NF-kappa-B inhibitor alpha (IκBα) and nuclear factor-κB (NF-κB). Specific inhibition of HO-1 cannot fully abolish its anti-inflammatory effect on the TNF-α-challenged cells. These results strongly suggested that CGA is a natural anti-inflammatory agent that can attenuate OS-induced inflammation and injury of intestinal epithelium via co-regulating the PI3K/Akt and IκBα/NF-κB signaling pathway.

## 1. Introduction

Oxidative stress (OS) is a physiological status characterized by an elevation of oxygen reactive species (ROS) in cells and tissues [1,2]. ROS are unstable and active oxygen-centered molecules containing unpaired valence-shell electrons. Under normal conditions, animals and humans possess effective capabilities of the generation and elimination of ROS. However, regardless of the endogenous sources, pollutants, radiation, diet, and lifestyle all can contribute to the elevation of intracellular ROS, leading to OS [3]. Importantly, increase in cellular ROS not only leads to frequent reactions with cell components, such as proteins, fats, and DNA, but also triggers various OS-related signaling pathways that are directly linked to tissue injury or the development of diseases [4,5,6,7]. Moreover, OS and inflammation are two closely interrelated and interdependent pathophysiological processes. ROS can initiate intracellular signaling cascades that upregulate the expression of proinflammatory genes [8]. On the other hand, inflammatory cells can secrete ROS and various immune mediators (e.g., cytokines and chemokines) leading to induction of OS and tissue damage at the site of inflammation [9].

The intestinal epithelium is the main site for nutrient absorption and provides a primary physical barrier against commensal and pathogenic microorganisms in the intestine. It has also been regarded as one of the major sources of ROS production, as the intestinal epithelium has inevitable exposure to exogenous substance and microbial pathogens [4]. Despite the protective barrier provided by the epithelial layer, ingested materials and pathogens can cause inflammation by activating the epithelium, polymorphonuclear neutrophils, and macrophages to produce inflammatory cytokines and other mediators that contribute further to OS. Importantly, various gastrointestinal diseases, including peptic ulcers, gastrointestinal cancers, and inflammatory bowel diseases, arise in part due to OS [4,10,11]. Thus, maintaining the ROS balance is urgently needed for the maintaining of intestinal health and for the treatment of associated intestinal diseases.

Chlorogenic acid (CGA), formed by esterification of caffeic acid and quinic acid, is one of the most abundant natural polyphenols present in coffee, fruits, and vegetables [12]. It also serves as a major active component in many Chinese medicinal herbs, such as *Eucommia*, *Honeysuckle*, and *Lonicera japonica* [13]. Previous studies indicated that CGA has multiple biological functions, including antioxidant, anti-inflammatory, and immunoprotective activities [14,15,16]. By far, many studies have been done to evaluate the potential health-promoting effects of CGA, indicating the beneficial effects of CGA on reducing the risk of metabolic syndrome or associated disorders, including diabetes, obesity and hypertension [17,18]. In addition, CGA was found to decrease the intestinal permeability and ameliorate intestinal injury in rat exposure to OS [19]. Our recent study also shows that CGA can attenuate weaning stress-induced intestinal injury in pigs by enhancing the activities of antioxidant enzymes and suppressing intestinal inflammatory cytokines [20]. However, the molecular mechanism of CGA action still remains unclear.

Diquat is a classic OS inducer, which is readily converted to a free radical in the presence of molecular oxygen, and subsequently produces superoxide anions and other redox products [21,22]. Therefore, to clarify the exact role of CGA and its mechanism of action, we explored the protective effect of CGA on intestinal epithelium in a porcine model exposure to OS induced by diquat [23]. We also investigated the protective potential of CGA against intestinal epithelium injury induced by diquat or tumor necrosis factor-α (TNF-α) in the porcine jejunal epithelial cell line IPEC-J2, to prove its effectiveness even when administered in vitro. Here, we report convincing evidence on the novel antioxidant effect of CGA and provide key insights into its potential mechanisms of action.

## 2. Materials and Methods

### 2.1. Reagents

Chlorogenic acid (Cat no. C3878, purity ≥ 95%) and diquat (Cat no. 45422) was purchased from Sigma (Sigma–Aldrich, Shanghai, China). Dulbecco’s Modified Eagle’s Medium and Ham’s F-12 Nutrient Mixture (DMEM/F12) medium and fetal bovine serum were obtained from HyClone (Logan UT, USA). TNF-α was purchased from RayBiotech (Norcross, GA, USA). LY294002 (Cat no. Z274722) and (E)3-[(4-methylphenyl)-sulfonyl]-2-propenenitrile (BAY11-7082) were purchased from Selleck (Selleckchem, Shanghai, China). Zn-protoporphyrin-IX (ZnPPIX, Cat no. 14483) was purchased from Cayman (Cayman Chemical, Beijing, China).

### 2.2. Animal Trial and Sample Collection

The OS was induced by using the diquat [23]. Twenty-four weaned pigs (Duroc × Landrace × Yorkshire), with an initial average body weight (BW) of 7.47 ± 0.50 kg, were randomly allotted to three groups (*n* = 8): (1) non-challenged control (CON; pigs fed with basal diet); (2) diquat-challenged control (diquat; pigs fed with basal diet and challenged by diquat); (3) diquat challenge + CGA treatment (DCGA; pigs fed with basal diet (Table S1) containing 1000 mg/kg CGA and challenged by diquat). The supplementary level of CGA to feed was based on our previous study [24]. Pigs were housed individually for 21 d in metabolism cages (0.70 × 1.50 m) and had free access to feed and water in an environmentally controlled house. On 15 d, pigs were either challenged by sterile saline or diquat (10 mg/kg BW) via intraperitoneal injection [23]. At the end of the trial, blood was collected from pigs after 12-h fasting. The serum samples were obtained by centrifugation at 3000× *g* for 15 min at 4 °C, and were subsequently stored at −20 °C for further analysis. Following blood collection, the pigs were euthanized with an intravenous injection of sodium pentobarbital (200 mg/kg body weight), and the abdomen was immediately opened to collect the jejunal segments. Briefly, about 2-cm segments of the middle jejunum were isolated, gently flushed with ice-cold phosphate buffer saline (PBS), and then preserved with ice-cold PBS for flow cytometry. Additionally, another 3-cm of segment was fixed in 4% paraformaldehyde solution for immunofluorescence and histological analyses. The mucosa samples were harvested by scraping the segment using a sterile glass slide, and were snap-frozen in liquid nitrogen and then stored at −80 °C until further analysis.

### 2.3. Cell Culture

The porcine jejunal epithelial cell line IPEC-J2 was obtained from American Type Culture Collection and cultured in DMEM/F12 medium supplemented with 10% fetal bovine serum, 100 IU/mL of penicillin, 100 mg/mL of streptomycin, 5 mg/mL epidermal growth factor, and 10 nM N-2-hydroxyethylpiperazine-N-2-ethane sulfonic acid, at 37 °C in a humidified atmosphere of 5% CO_2_. All cells were washed with PBS (pH 7.2–7.4) before treatment with CGA, diquat, or TNF-α. After growing in 80% confluence in 6-well plates, the cells (pretreated with 100 µM CGA for 6 h) were exposed to 100 µM diquat for 3 h, or the cells (pretreated with 50 µM CGA for 6 h) were exposed to 50 ng/mL TNF-α for 3 h. The culture supernatants and pellets were collected and stored in −80 °C for further analysis. Cell viability was analyzed by using the cell counting kit-8 with a microplate reader (SpectraMax M2, Molecular Devices, Sunnyvale, CA, USA).

### 2.4. Flow Cytometric Assays

Cellular apoptosis was assessed by using fluorescein isothiocyanate (FITC)-, Alexa Fluor^®^647-conjugated Annexin V with propidium iodide (PI) staining assay (Biolegend; 640914 and 610912) according to the manufacturer’s instructions. Briefly, cells were harvested and resuspended in 100 μL/10^6^ cells of 1 × binding buffer. After that, 2 μL of Annex V per test were added and incubated for 20 min in the dark on ice. Subsequently, 400 μL 1× binding buffer and 1 μL PI (1 mg/mL) was added into the reaction tubes and then mixed thoroughly. The apoptotic cells were immediately measured by flow cytometry and analyzed with FlowJo software (FlowJoLLC).

### 2.5. Immunofluorescence Assays

The distribution of the tight junction protein (claudin-1) in the intestinal epithelium and IPEC-J2 cells was determined by using immunofluorescence analysis. Briefly, the jejunal segments were deparaffinized and then subjected to antigen retrieval by ethylene diamine tetraacetic acid (1 M, pH 9.0). Sections were blocked with 3% bovine serum albumin and incubated with rabbit anti-claudin-1 antibody (1:100 dilution; Abcam plc., Cambridge, UK) overnight at 4 °C. The sections were washed three times with PBS (pH 7.4), and then treated by goat anti-rabbit IgG-FITC secondary antibody (Beijing Zhongshan Golden Bridge Biotechnology Co., Ltd., Beijing, China), followed by incubation at room temperature for 50 min in the dark. Sections were then washed with PBS (PH 7.4), and the nuclei were stained with 4’,6-diamidino-2-phenylindole (DAPI) for 10 min at room temperature. For in vitro studies, the cultured cells were fixed with 4% paraformaldehyde solution, and then 0.5% Triton X-100 was used for the permeation of the cells. After the successive incubation with the first and second antibodies, DAPI was added for the generation of fluorescence. The slides were visualized under a laser scanning confocal microscope (FV1000; Olympus Corporation, Tokyo, Japan).

### 2.6. Intracellular ROS Assays

The content of intracellular ROS was measured by using the ROS detection kit (ab113851, Abcam). Briefly, after specific experimental treatment, the culture medium was discarded and then incubated with 10 μL of 2′,7′-Dichlorodihydrofluorescein diacetate for 1 h at 37 °C. Cells were washed thrice with PBS and then resuspended in PBS. Finally, the intracellular ROS level was examined with a NanoDrop spectrophotometer (Beckman Coulter DU800; Beckman Coulter Inc., Fullerton, CA, USA) at the excitation wavelength of 500 (500 ± 15 nm) and the optimal emission wavelength of 525 (530 ± 20 nm).

### 2.7. Western Blotting Analysis

Proteins were extracted from IPEC-J2 cells by using the RIPA lysis buffer. Protein concentrations were determined by using the bicinchoninic acid assay kit (ab102536, Abcam) and Nano-Drop ND 2000c Spectrophotometer (Thermo Scientific, Waltham, MA, USA). Equal amounts of proteins were resolved through sodium dodecyl sulfate polyacrylamide gel electrophoresis on 10% polyacrylamide gels, and then transferred onto polyvinylidene fluoride (PVDF) membrane (Millipore, Eschborn, Germany) with a wet Trans-Blot system (Bio-Rad, Richmond, CA, USA). After blocked with 0.1% Tris-buffered saline (TBS/T) supplemented with 5% bovine serum albumin for 1 h at room temperature, the membranes were incubated in specific primary antibodies [protein kinase B (Akt, CST, 9272), pAkt (CST, 9271), nuclear factor erythroid-derived-related factor 2 (Nrf2, Cayman, ab137550), pNrf2 (Cayman, ab76026), heme oxygenase-1 (HO-1, Cayman, ab13248), nuclear factor-κBp65 (NF-κBp65, CST, 6956), pNF-κBp65 (CST, 3033), NF-kappa-B inhibitor alpha (IκBα, CST, 4814), pIκBα (CST, 8219) and β-actin (Santa Cruz, 47778)] at 4 °C overnight (1:1000 dilution). The PVDF membranes were washed with TBS/T and then incubated with second antibodies for 1 h at room temperature. The second goat anti-rabbit and goat anti-mouse antibodies conjugated to horseradish peroxidase (Santa Cruz, sc-2030 and sc-2031) was used (1:3000 dilution). Signal densities of the immunoblotting image were determined using ClarityTM Western ECL Substrate (Bio-Rad, Hercules, CA, USA) on a ChemiDocTM XRS+ Imager System (Bio-Rad).

### 2.8. RNA Extraction and QPCR

Total RNA was extracted from the cells by using the Trizol reagent (TaKaRa, Dalian, China) following the manufacturer’s instructions. The purity and concentration of all RNA samples were checked by a NanoDrop spectrophotometer (Beckman Coulter DU800; Beckman Coulter Inc., Fullerton, CA, USA) at 260 and 280 nm. Reverse transcription was performed using the PrimeScripte RT reagent kit with gDNA Eraser (TaKaRa, Dalian, China). All primers were synthesized commercially by Sangon Biotech Limited and were shown in Table S2. The expression levels of genes were detected by using the CFX-96 Real-Time PCR Detection System (Bio-Rad Laboratories, Richmond, CA, USA) and SYBR Premix Ex Taq II (Tli RNaseH Plus) reagents (TaKaRa, Dalian, China). The glyceraldehyde-3-phosphate dehydrogenase was used as the house-keeping gene to normalize the expression of target genes. Relative expression was calculated using the comparative cycle threshold (2^−ΔΔCt^) method [25].

### 2.9. Biochemical Analysis

Serum xylose concentration was determined by using the xylose detection kit (Jiancheng Bioengineering Institute, Nanjing, China) with a spectrophotometer (Beckman Coulter DU-800; Beckman Coulter, Inc.). Tissues or cells were homogenized in ice with RIPA lysis buffer. Antioxidant-related kits from Nanjing Jiancheng Bioengineering Institute were used to determine the content of the malondialdehyde (MDA), superoxide dismutase (SOD), catalase (CAT), glutathione peroxidase (GSH-Px), and total antioxidative capability (T-AOC) according to the manufacturers’ instructions.

### 2.10. Statistical Analysis

All results are expressed as the means and standard deviation. Data were subjected to one-way analysis of variance (ANOVA) followed by Tukey’s multiple-range test to determine significant differences among the treatments at *p* < 0.05 with SAS 9.0 software (SAS Inst. Inc., Cary, NC, USA).

## 3. Results

### 3.1. CGA Attenuates OS-Induced Inflammation and Intestinal Epithelium Injury in Weaned Pigs

**Figure 1:**
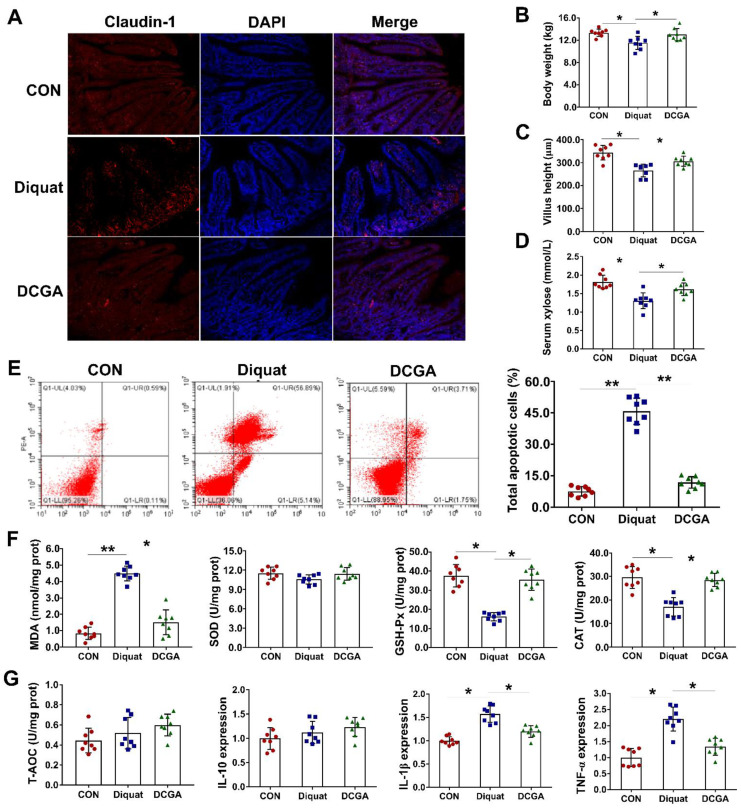
Chlorogenic acid (CGA) attenuates oxidative stress-induced inflammation and intestinal epithelium injury in weaned pigs. (**A**) Localization of the tight-junction protein claudin-1 by immunofluorescence staining. (**B**) Body weight. (**C**) Villus height of the jejunum in weaned pigs upon oxidative stress. (**D**) Serum concentrations of xylose. (**E**) Apoptosis of the intestinal epithelial cells. In each diagram, Q1-UL represents the percentage of nonviable and necrotic cells, Q1-UR represents the percentage of late apoptotic cells, Q1-LR represents the percentages of early apoptotic cells, and Q1-LL represents the percentage of live cells. (**F**) Abundances of malondialdehyde (MDA) and antioxidant enzymes in the intestinal mucosa. (**G**) Abundance of total antioxidative capability (T-AOC) and expressions of IL-10, IL-beta and TNF-alpha, respectively, in the intestinal mucosa. CON, pigs fed with basal diet; diquat, pigs fed with basal diet and challenged by diquat; DCGA, pigs fed with basal diet containing 1000 mg/kg CGA and challenged by diquat (*n* = 8). * *p* < 0.05, ** *p* < 0.01.

Our previous study showed that CGA could enhance the intestinal antioxidant and anti-inflammatory capacities of weaned pigs [20]. To further explore the protective effect of CGA, weaned pigs were treated with or without CGA, and subsequently exposed to OS (diquat challenge) in the present study. Results showed that diquat challenge significantly decreased the body weight and led to injury of the intestinal epithelium, as indicated by decreased villus height and reduced absorption of xylose (Figure 1A–D). Immunofluorescence also showed that diquat challenge decreased the abundance of the tight-junction protein (claudin-1). However, CGA supplementation not only improved the integrity of the intestinal epithelium, but also significantly reduced the apoptosis of the intestinal epithelial cells upon diquat challenge (Figure 1E). Interestingly, CGA supplementation elevated the activity of antioxidant enzymes, such as the GSH-Px and CAT, but significantly decreased the content of MDA and inflammatory cytokines, such as the interleukin-1β (IL-1β) and TNF-α in the intestinal mucosa upon diquat challenge (Figure 1F,G).

### 3.2. Protective Effect of CGA on IPEC-J2 Cell Exposure to OS

**Figure 2:**
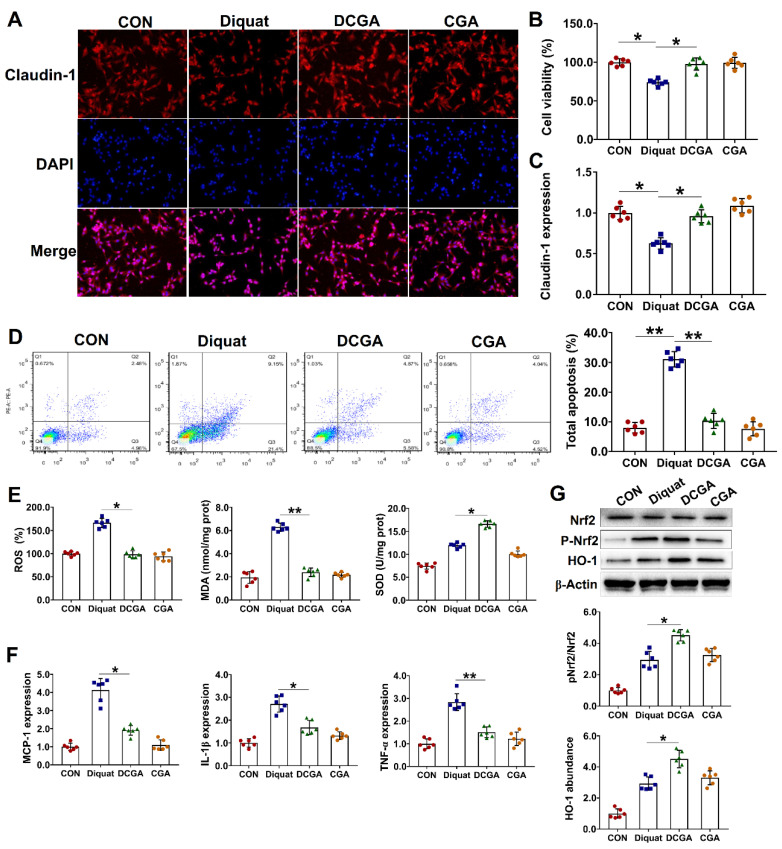
Protective effect of chlorogenic acid (CGA) on IPEC-J2 cells exposure to oxidative stress. (**A**) Localization of the tight-junction protein claudin-1 by immunofluorescence staining. IPEC-J2 cells were plated in 24-well plates at a density of 1 × 10^5^ cells/well and pretreated by CGA (100 µM) for 6 h, and the cells were then challenged by diquat (100 µM) for 3 h (*n* = 6). (**B**) Assays of cell viability. (**C**) Expression level of claudin-1. (**D**) Analysis of cell apoptosis by flow cytometric assays. In each diagram, Q1 represents the percentage of nonviable and necrotic cells, Q2 represents the percentage of late apoptotic cells, Q3 represents the percentages of early apoptotic cells, and Q4 represents the percentage of live cells. (**E**) Intracellular contents of oxygen reactive species (ROS), malondialdehyde (MDA), and superoxide dismutase (SOD). (**F**) qPCR analysis of the expression levels of critical inflammation-related molecules. (**G**) Western-blot analysis of the protein abundances of nuclear factor erythroid-derived-related factor 2 (Nrf2) and heme oxygenase-1 (HO-1). CON, cells without being treated; Diquat, cells were only treated by 100 µM diquat; CGA, cells were only treated by 100 µM CGA; DCGA, cells were pretreated by 100 µM CGA for 6 h and were then treated by 100 µM diquat for 3 h. * *p* < 0.05, ** *p* < 0.01.

We next investigated the protective potential of CGA against diquat-induced OS and injury in IPEC-J2. The IPEC-J2 cells were pretreated with CGA and then challenged by diquat. Results showed that diquat challenge significantly decreased the cell viability and the abundance of tight-junction proteins (Figure 2A–C). Moreover, the diquat challenge elevated the apoptosis rate in the IPEC-J2 cells. However, CGA pretreatment not only improved the integrity of the cells, but also reduced the number of apoptotic cells upon diquat challenge (Figure 2D). CGA had no influences on cellular ROS and antioxidant enzyme in the IPEC-J2 cells without being diquat-challenged. However, CGA pretreatment significantly reduced the cellular ROS and MDA content, but increased the SOD content in the diquat-challenged cells (Figure 2E). As the OS and inflammation are two closely interrelated and interdependent pathophysiological processes [19,20], we next investigated the expression levels of several critical molecules involved in the inflammatory response. As shown in Figure 2F, diquat challenge significantly elevated the expression levels of monocyte chemotactic protein-1 (MCP-1), IL-1β, and TNF-α in the IPEC-J2 cells. However, CGA pretreatment significantly down-regulated their expressions in the diquat-challenged cells. Nrf2 and HO-1 are two critical regulators involved in the cellular antioxidative responses [16,26]. We found that CGA pretreatment significantly elevated their protein abundance in the diquat-challenged cells (Figure 2G).

### 3.3. CGA Attenuates OS-Induced Injury in IPEC-J2 Cells via phosphatidylinositol-3-kinase (PI3K)/Akt Signaling

**Figure 3:**
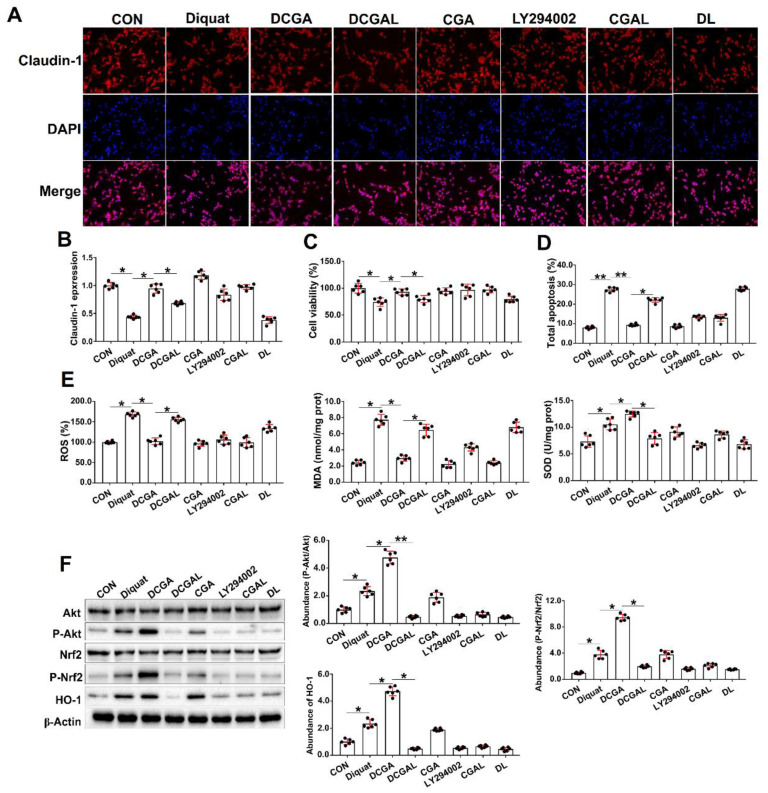
Chlorogenic acid (CGA) attenuates oxidative stress-induced injury in IPEC-J2 cells via phosphatidylinositol-3-kinase (PI3K)/protein kinase B (Akt) signaling. (**A**) Localization of the tight-junction protein claudin-1 by immunofluorescence staining. IPEC-J2 cells were plated in 24-well plates at a density of 1 × 10^5^ cells/well (*n* = 6). (**B**) qPCR analysis of the expression levels of claudin-1. (**C**) Assays of cell viability. (**D**) Analysis of cell apoptosis by flow cytometric assays. (**E**) Intracellular contents of oxygen reactive species (ROS), malondialdehyde (MDA), and superoxide dismutase (SOD). (**F**) Western-blot analysis of critical signaling proteins involved in the PI3K/Akt pathway. CON, cells without being treated; Diquat, cells were only treated by 100 µM diquat; CGA, cells were only treated by 100 µM CGA; DCGA, cells were pretreated by 100 µM CGA for 6 h and were then treated by 100 µM diquat for 3 h. DCGAL, cells were pretreated by 10 µM LY294002 for 1 h and were then treated by 100 µM CGA and 100 µM diquat; LY294002, cells were only treated by 10 µM LY294002; CGAL, cells were pretreated by 10 µM LY294002 for 1 h and were then treated by 100 µM CGA; DL, cells were pretreated by 10 µM LY294002 for 1 h and were then treated by 100 µM diquat. * *p* < 0.05, ** *p* < 0.01.

Activation of the PI3K/Akt signaling results in the translocation of Nrf2 and transcription of genes coding a series of cellular antioxidant enzymes [27]. To verify the effectiveness of CGA and the potential mechanisms of action, a specific inhibitor (LY294002) of the PI3K/Akt signaling was used in this study. We show that treating the cells with LY294002 alone had no influences on cell viability, tight junction protein, and cell apoptosis in the diquat-challenged cells. However, pretreatment of the cells with LY294002 abolished the protective effect of CGA on diquat-challenged cells, as indicated by the decreased abundance of tight junction protein and cell viability, and elevated apoptosis rate (Figure 3A–D). Moreover, LY294002 abolished the antioxidant capacity of CGA, as indicated by the increased ROS and MDA content, and the decreased SOD content in the diquat-challenged cells (Figure 3E). We also investigated the abundances of critical signaling proteins involved in the PI3K/Akt signaling pathway. As expected, CGA not only elevated the abundances of the phosphorylated Akt and Nrf2, but also elevated the protein abundance of HO-1 in the diquat-challenged cells. However, the LY294200 (combined with CGA or alone) significantly decreased their abundances in the IPEC-J2 upon diquat challenge (Figure 3F).

### 3.4. CGA-Regulated HO-1 Expression Suppresses IκBα/NF-κB Signaling in IEPC-J2 Cell Exposure to OS

**Figure 4:**
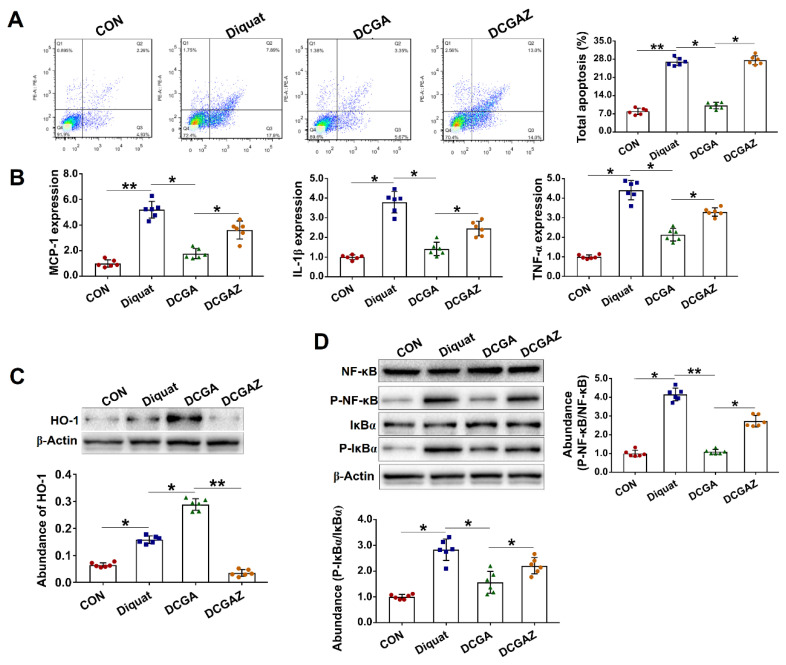
Chlorogenic acid (CGA)-regulated heme oxygenase-1 (HO-1) expression suppresses NF-kappa-B inhibitor alpha (IκBα)/nuclear factor-κB (NF-κB) signaling in IEPC-J2 cell exposure to oxidative stress. (**A**) Analysis of cell apoptosis by flow cytometric assays. In each diagram, Q1 represents the percentage of nonviable and necrotic cells, Q2 represents the percentage of late apoptotic cells, Q3 represents the percentages of early apoptotic cells, and Q4 represents the percentage of live cells. IPEC-J2 cells were plated in 24-well plates at a density of 1 × 10^5^ cells/well (*n* = 6). (**B**) qPCR analysis of the expression levels of critical inflammation-related molecules. (**C**) Western-blot analysis of the abundance of heme oxygenase-1 (HO-1). (**D**) Western-blot analysis of critical signaling proteins involved in the IκBα/NF-κB pathway. CON, cells without being treated; diquat, cells were treated by 100 µM diquat; DCGA, cells were pretreated by 100 µM CGA for 6 h and were then treated by 100 µM diquat for 3 h; DCGAZ, cells were pretreated by 10 µM Zn-protoporphyrin-IX (ZnPPIX, the specific inhibitor of HO-1) and were then treated by 100 µM CGA and 100 µM diquat. * *p* < 0.05, ** *p* < 0.01.

HO-1 is one of the downstream targets of the Nrf2 and acts as a critical regulator of the cellular stress response and inflammation [28]. We found that treating the cells with a specific inhibitor of HO-1 (ZnPPIX) abolished the protective effect of CGA in the diquat-challenged cells, as indicated by increased apoptosis rate and elevated expressions of inflammatory cytokines (Figure 4A,B). IκBα and NF-κB are two critical inflammation-associated signaling proteins that are regulated by various upstream regulators, including the HO-1 [28,29]. In the present study, CGA treatment significantly reduced the protein abundances of phosphorylated IκBα and NF-κB in the diquat-challenged cells. However, specific inhibiting of the HO-1 abolished the inhibitory effects of CGA on the two critical signaling proteins (Figure 4C,D).

### 3.5. CGA Attenuates TNF-α Induced Injury in IPEC-J2 Cells via Direct Suppressing of the IκBα/NF-κB Signaling

**Figure 5:**
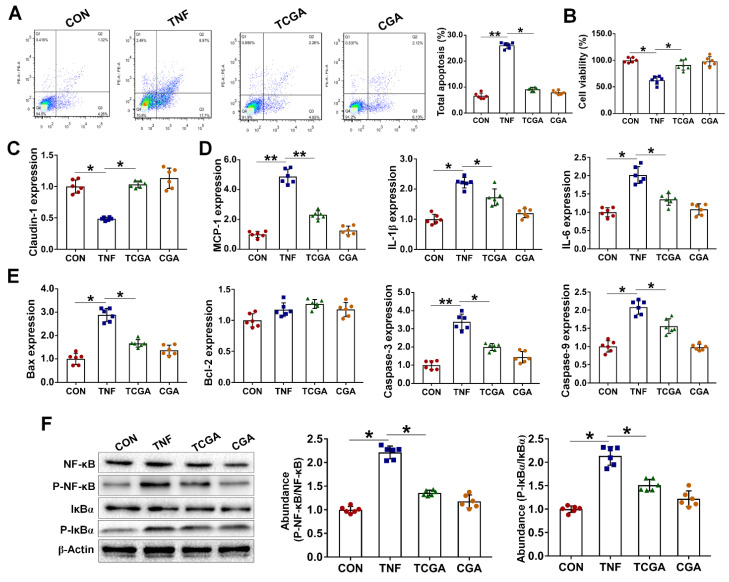
Chlorogenic acid (CGA) attenuates tumor necrosis factor-α (TNF-α) induced inflammation and injury in IPEC-J2 cells. (**A**) Analysis of cell apoptosis by flow cytometric assays. In each diagram, Q1 represents the percentage of nonviable and necrotic cells, Q2 represents the percentage of late apoptotic cells, Q3 represents the percentages of early apoptotic cells, and Q4 represents the percentage of live cells. IPEC-J2 cells were plated in 24-well plates at a density of 1 × 10^5^ cells/well and pretreated by CGA (50 µM) for 6 h, and the cells were then challenged by TNF-α (50 ng/mL) for 3 h (*n* = 6). (**B**) Assays of cell viability. (**C**) qPCR analysis of the expressions of claudin-1. (**D**) Expressions of critical inflammation-related molecules. (**E**) Expressions of critical apoptosis-related proteins. (**F**) Western-blot analysis of critical signaling proteins involved in the NF-kappa-B inhibitor alpha (IκBα)/ nuclear factor-κB (NF-κB) pathway. CON, cells without being treated; TNF, cells were only treated by 50 ng/mL TNF-α; TCGA, cells were pretreated by 50 µM CGA for 6 h and were then treated by 50 ng/mL TNF-α for 3 h; CGA, cells were only treated by 50 µM CGA. * *p* < 0.05, ** *p* < 0.01.

**Figure 6:**
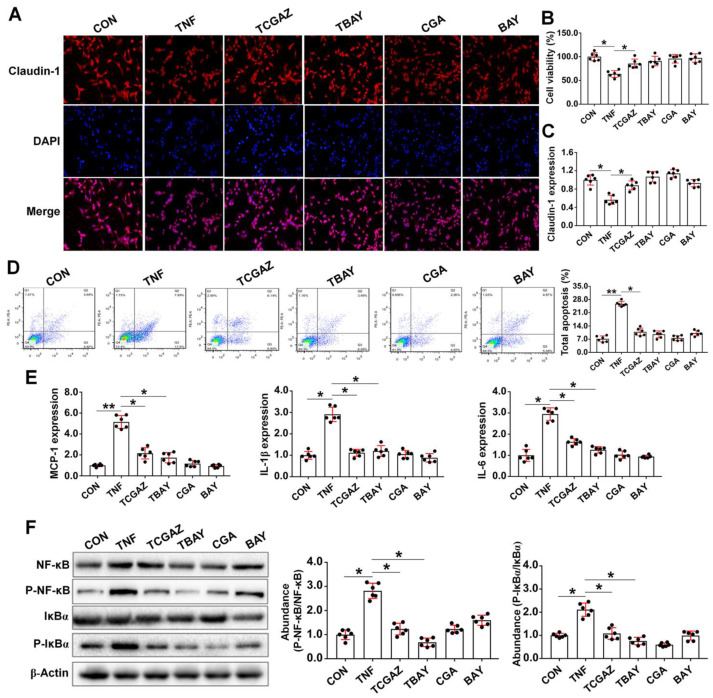
Chlorogenic acid (CGA) suppresses tumor necrosis factor-α (TNF-α) induced inflammation and injury that is independent on the phosphatidylinositol-3-kinase (PI3K)/protein kinase B (Akt)/ heme oxygenase-1 (HO-1) pathway. (**A**) Localization of the tight-junction protein claudin-1 by immunofluorescence staining. IPEC-J2 cells were plated in 24-well plates at a density of 1 × 10^5^ cells/well (*n* = 6). (**B**) Assays of cell viability. (**C**) qPCR expressions of claudin-1. (**D**) Analysis of cell apoptosis by flow cytometric assays. In each diagram, Q1 represents the percentage of nonviable and necrotic cells, Q2 represents the percentage of late apoptotic cells, Q3 represents the percentages of early apoptotic cells, and Q4 represents the percentage of live cells. (**E**) Expressions of critical inflammation-related molecules. (**F**) Western-blot analysis of critical signaling proteins involved in the NF-kappa-B inhibitor alpha (IκBα)/nuclear factor-κB (NF-κB) pathway. CON, cells without being treated; TNF, cells were treated by 50 ng/mL TNF-α; TCGAZ, cells were pretreated by 10 µM Zn-protoporphyrin-IX (ZnPPIX, the specific inhibitor of HO-1) for 1 h and were then treated by 50 µM CGA and 50 ng/mL TNF-α; TBAY, cells were pretreated by 20 µM (E)3-[(4-methylphenyl)-sulfonyl]-2-propenenitrile (BAY11-7082, the specific NF-κB inhibitor) and were then treated by 50 ng/mL TNF-α; CGA, cells were only treated by 50 µM CGA; BAY, cells were only treated by 20 µM BAY11-7082. * *p* < 0.05, ** *p* < 0.01.

As the OS and inflammation are two closely interrelated pathophysiological processes, we next investigated the protective effect of CGA on inflammation-induced injury in the IPEC-J2 cells upon TNF-α challenge. We show that TNF-α challenge led to injury of the cells, as indicated by increases in cell apoptosis and expressions of a critical inflammation- or apoptosis-related molecules, such as the MCP-1, IL-1β, IL-6, Bax, and caspase3/9 (Figure 5A–E). However, CGA treatment significantly attenuated injury and inflammatory responses in the IPEC-J2 cells exposure to TNF-α. Importantly, CGA significantly decreased the protein abundances of phosphorylated IκBα and NF-κB in the TNF-α-challenged cells (Figure 5F). Moreover, specific inhibiting of the NF-κB (using BAY11-7082) also attenuated injury and inflammatory responses in cells exposure to TNF-α (Figure 6). However, specific inhibiting of the HO-1 did not abolish the protective effect of CGA on the TNF-α-challenged cells.

## 4. Discussion

The gastrointestinal (GI) tract is a key source of ROS generation, as the gastrointestinal mucosa is constantly challenged by diet-derived oxidants, mutagens, and carcinogens as well as by the endogenously generated ROS [30]. ROS are key signaling molecules that play an important role in the progression of many inflammatory disorders [9]. Evidence is accumulating to show that various GI pathological conditions, including gastroduodenal ulcers, GI malignancies, and inflammatory bowel disease, arise in part from OS [4,31]. Diquat, a classic OS inducer, can be readily converted to a free radical in the presence of molecular oxygen and subsequently generates superoxide anions and other redox products. [21,22]. In the present study, diquat challenge induced intestinal inflammation and impaired the intestinal epithelial functions (e.g., increased apoptosis of the intestinal epithelial cells) both in vivo and in vitro. However, CGA not only reduced generation of intracellular ROS, but also attenuated the OS-induced inflammation and injury, indicating the prominent antioxidative property of the natural polyphenols.

Although the free radicals are continuously generated, the antioxidant defense system in the body can help to defend against the harmful effects of ROS. Currently, several mechanisms mediating protection against OS are documented [5]. Amongst these mechanisms, the PI3K/Akt signaling pathway has been considered the most important one in the regulation of OS, and activation of this signaling pathway results in the translocation of Nrf2 and transcription of genes coding a series of cellular antioxidant enzymes [32,33]. In the present study, CGA treatment attenuated OS-induced injury and significantly elevated the abundances of phosphorylated Akt and Nrf2 in the IPEC-J2 cells. As expected, the intracellular SOD content was significantly elevated in the diquat-challenged cells after CGA treatment. SOD is one of the major antioxidant enzymes for mammalian animals, including the human. Currently, three distinct isoforms of SOD have been identified and characterized in mammals: copper–zinc superoxide dismutase (Cu/ZnSOD), manganese superoxide dismutase (MnSOD), and extracellular superoxide dismutase (ECSOD), and they elicit similar functions [34,35]. However, treatment of the cells with a specific PI3K inhibitor abolished the protective effect of CGA against the OS, as indicated by increased cell apoptosis and ROS generation. The result suggested that CGA may attenuate the OS-induced injury via regulating the PI3K/Akt signaling pathway.

Activation of the Nrf2 represents a cellular defense response against OS. Previous study indicated that Nrf2 activates a transcription of a wide variety of genes involved in redox balance, detoxification of xenobiotics, metabolism, and inflammation [36]. HO-1, a rate-limiting enzyme of heme degradation, is an endogenous, cytoprotective enzyme that is activated by Nrf2 during cellular OS [37]. Importantly, numerous studies indicated that HO-1 plays a critical role in maintain antioxidant/oxidant homeostasis and inducing immunomodulatory, anti-inflammatory, and antiapoptotic effects [37,38,39]. In the present study, CGA significantly elevated the abundances of phosphorylated Nrf2 and HO-1 in the diquat-challenged cells, and attenuated its inflammatory responses as indicated by decreases in cell apoptosis, secretion of inflammatory cytokines, and abundances of phosphorylated IκBα and NF-κB. However, treating the cells with a specific HO-1 inhibitor abolished its anti-inflammatory effect. Previous studies indicated that Nrf2/HO-1 downregulates the function of antigen-presenting cells, polarizes macrophages into anti-inflammatory phenotype (M2), and reduces detrimental immune activation [40,41,42]. Moreover, Nrf2/HO-1 and its metabolites may also modulate humoral immunity thus reducing the production of autoantibodies [43,44]. These results suggested that CGA could suppress the OS-induced inflammation and injury in the intestinal epithelium through indirect inhibition of the IκBα/NF-κB signaling pathway activation by promoting the expression of HO-1 via PI3K/Akt signaling pathway.

To further study the effect of CGA on the activation of IκBα/NF-κB signaling pathway, we also explored the protective effect of CGA on cellular inflammation that is independent on OS in the IPEC-J2 cells. TNF-α is a classic pro-inflammatory cytokine that can mediate host inflammatory response to various pathogens [45]. Importantly, overproduction of TNF-α not only increases the permeability of intestinal epithelium, but also induces apoptosis via the intrinsic mitochondrial apoptotic pathway [46,47]. In the present study, we found that CGA can exert a similar role to that of specific NF-κB inhibitor (BAY11-7082) and both can attenuate TNF-α induced inflammation and injury in the IPEC-J2 cells (as indicated by the decreases in cell apoptosis and expressions of critical inflammation-related molecules, such as the IκBα and NF-κB). However, specific inhibition of the HO-1 cannot fully abolish its anti-inflammatory effects, indicating that CGA can suppress the inflammation and injury of intestinal epithelium by inhibiting the activation of IκBα/NF-κB signaling pathway that is independent on the PI3K/Akt-Nrf2 signaling pathway.

## 5. Conclusions

**Figure 7:**
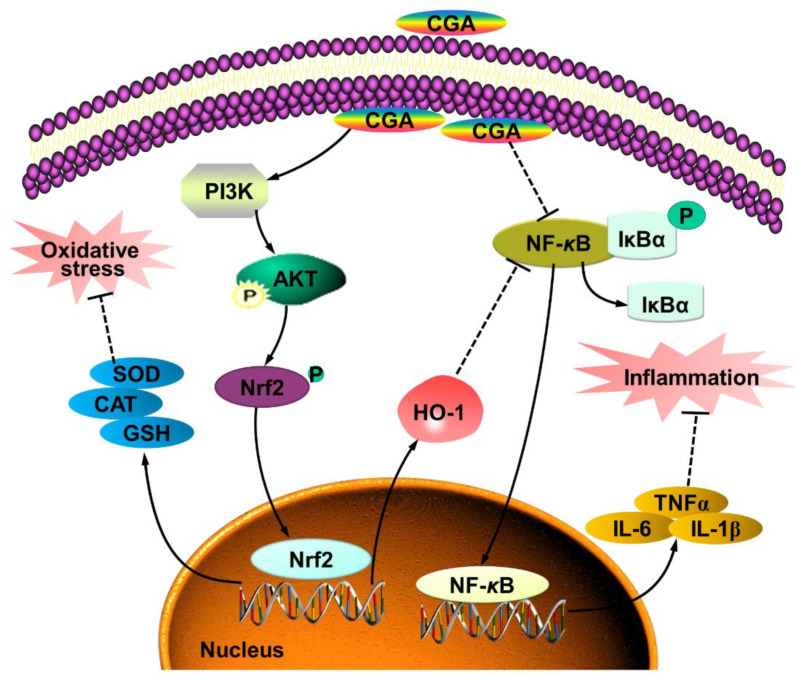
Model of proposed mechanism of chlorogenic acid (CGA)-attenuated intestinal inflammation and injury induced by oxidative stress. CGA protects the intestinal epithelium against oxidative stress-induced injury via regulating the phosphatidylinositol-3-kinase (PI3K)/protein kinase B (Akt)/ nuclear factor erythroid-derived-related factor 2 (Nrf2) signaling pathway; CGA attenuates the intestinal inflammation by co-regulating the PI3K/Akt/heme oxygenase-1 (HO-1) and NF-kappa-B inhibitor alpha (IκBα)/nuclear factor-κB (NF-κB) signaling pathway.

In summary, the present study provides novel insights into the inhibiting effects of CGA on OS-induced intestinal inflammation and epithelium dysfunction. The mechanisms of action might be associated with co-regulating the PI3K/Akt and IκBα/NF-κB signaling pathway (Figure 7). The antioxidant and anti-inflammatory properties of CGA should make it an attractive candidate for the prevention or treatment of various OS-induced injuries and diseases.

## Data Availability

Data presented in this study are presented in the article and supplementary material.

## Supplementary Materials

The following are available online at https://www.mdpi.com/article/10.3390/antiox10121915/s1, Table S1: Ingredient composition and nutrient levels of basal diets (air-dry basis, %), Table S2: Primers used for real-time quantitative PCR.
