# Supplementary material for: Chlorogenic Acid Attenuates Oxidative Stress-Induced Intestinal Epithelium Injury by Co-Regulating the PI3K/Akt and IκBα/NF-κB Signaling
Source: Antioxidants (Basel). 2021 Nov 29;10(12):1915. doi: 10.3390/antiox10121915 (PMC8750628; doi:10.3390/antiox10121915)
Supplement: Supplementary file 1 [file antioxidants-10-01915-s001.zip › antioxidants-1463395-SI.pdf]

Table S1. Ingredient composition and nutrient levels of basal diets (air-dry basis, %).

Ingredient	%	Nutrient concentrations[1]	%
Corn	28.00	CP	20.36
Extruded corn	28.00	ME (MJ/kg)	14.83
Soybean meal	10.00	Ca	0.82
Extruded soybean	7.00	Total P	0.61
Fish meal	5.00	Available P	0.43
Whey powder	7.00	Lysine	1.37
Soybean protein concentrate	8.00	Methionine	0.45
Soybean oil	2.16	Methionine + cystine	0.74
Sucrose	2.50	Threonine	0.81
Limestone	0.70	Tryptophan	0.21
Dicalcium phosphate	0.45		
Salt	0.30		
L-lysine HCl	0.28		
DL-Methionine	0.12		
L-Threonine	0.04		
Choline chloride	0.10		
Vitamin premix[2]	0.05		
Mineral premix[3]	0.30		

[1]Values are calculated

[2]The premix provides following per kilogram of diet: Vitamin A, 6000 IU; Vitamin D_3, 400 IU; Vitamin E, 10 IU; Vitamin K_3, 2 mg; Vitamin B_1, 0.8 mg; Vitamin B_2, 6.4 mg; Vitamin B_6, 2.4 mg; Vitamin B_{12}, 12 μg; folic acid, 0.2 mg; nicotinic acid, 14 mg; D-pantothenic acid, 10 mg

[3]The premix provides following per kilogram of diet: Fe (as ferrous sulfate), 130 mg; Cu (as copper sulfate), 80 mg; Mn (as manganese sulfate), 60 mg; Zn (zinc sulfate), 120 mg; I (potassium iodide), 0.3 mg; Se (as sodium selenite), 0.35 mg

Table S2. Primers used for real-time quantitative PCR[1].

Gene	Accession NO.	Primer sequences[2] (5'-3')	Size,bp
TNF-α	NM_214022	F: TTCCAGCTGGCCCCTTGAGC<br>R: GAGGGCATTGGCATACCCAC	146
IL-1β	NM_ 214055.1	F: CCTCTCCAGCCAGTCTTCAT<br>R: GCCATCAGCCTCAAATAACAG	126
IL-6	XM- 214399	F: TTCACCTCTCCGGACAAAAC<br>R: TCTGCCAGTACCTCCTTGCT	122
IL-10	NM- 214041.1	F: CGGCGCTGTCATCAATTTCTG<br>R: CCCCTCTCTTGGAGCTTGCTA	89
MCP-1	NM- 214214.1	F: ATTCTCCAGTCACCTGCTGC<br>R: TGCTGGTGACTCTTCTGTAGC	84
claudin-1	NM-001244539.1	F: ATTTCAGGTCTGGCTATCTTAGTTGC<br>R: AGGGCCTTGGTGTTGGGTAA	214
Bax	XM_ 013998624.2	F: GACGCTGGACTTCCTTCGAG<br>R: GTGGCCCGAGAGAGGTTTATT	334
Bcl-2	XM_021099593.1	F: GCTACTTACTGCCAAAGGGA<br>R: TTCAGGCGGAGCTGTAAGAG	161
Caspase-3	NM_214131.1	F: GGAATGGCATGTCGATCTGGT<br>R: ACTGTCCGTCTCAATCCCAC	351
Caspase-9	XM_013998997.2	F: AATGCCGATTTGGCTTACGT<br>R: CATTTGCTTGGCAGTCAGGTT	195
GAPDH	NM_001206359.1	F: TCGGAGTGAACGGATTTGGC<br>R: TGCCGTGGGTGGAATCATAC	147

[1] *TNF*: Tumor necrosis factor, *IL*: Interleukin, *MCP-1*: Monocyte chemoattractant protein-1, *Bax*: B-cell lymphoma-2-associated X protein, *Bcl-2*: B-cell lymphoma-2, *GAPDH*: glyceraldehyde-3-phosphate dehydrogenase.

[2]F: forward, R: reverse.
